# Effect of salt stress on ion concentration, proline content, antioxidant enzyme activities and gene expression in tomato cultivars

**DOI:** 10.1093/aobpla/plw055

**Published:** 2016-10-26

**Authors:** Charfeddine Gharsallah, Hatem Fakhfakh, Douglas Grubb, Faten Gorsane

**Affiliations:** 1 Laboratory of Molecular Genetics, Immunology and Biotechnology, Faculty of Sciences of Tunis, University of Tunis ElManar, Tunis 2092, Tunisia; 2 Faculty of Sciences of Bizerte, University of Carthage, Zarzouna 7021, Tunisia; 3 Department of Molecular Signal Processing, Leibniz Institute of Plant Biochemistry, Weinberg 3, Halle, 06120 Saale, Germany

**Keywords:** Antioxidant enzymes, gene expression, ion contents, qPCR, salt stress, Tunisian tomato genotypes

## Abstract

Salinity is a constraint limiting plant growth and productivity of crops throughout the world. Understanding the mechanism underlying plant response to salinity provides new insights into the improvement of salt tolerance-crops of importance. In the present study, we report on the responses of twenty cultivars of tomato. We have clustered genotypes into scale classes according to their response to increased NaCl levels. Three local tomato genotypes, representative of different saline scale classes, were selected for further investigation. During early (0 h, 6 h and 12 h) and later (7 days) stages of the response to salt treatment, ion concentrations (Na^+^, K^+ ^ and Ca^2+^), proline content, enzyme activities (catalase, ascorbate peroxidase and guiacol peroxidase) were recorded. qPCR analysis of candidate genes *WRKY* (8, 31and 39), *ERF* (9, 16 and 80), *LeNHX* (1, 3 and 4) and *HKT* (class I) were performed. A high K^+^, Ca^2 +^and proline accumulation as well as a decrease of Na^+ ^ concentration-mediated salt tolerance. Concomitant with a pattern of high-antioxidant enzyme activities, tolerant genotypes also displayed differential patterns of gene expression during the response to salt stress.

## Abstract (precis)

Understanding plant response to salinity, one of the major abiotic stresses, provides insights into the improvement of tomato salt tolerance. This work focuses on the responses of tomato cultivars to salt stress. Genotypes, representative of content and enzyme activities. QPCR analysis of WRKY, ERF, LeNHX and HKT genes was also performed. A high K+, Ca2+ and proline accumulation as well as a decrease in Na+ concentration mediated salt tolerance. Concomitant with a pattern of high antioxidant enzyme activities, tolerant genotypes also displayed differential patterns of gene expression.

## Introduction

High salinity is an important abiotic stress that reduces crop productivity in arid and semi-arid regions of the world (Foolad 2007). It is estimated that, worldwide, 800 million ha of land and 32 million ha of agricultural land are salt-affected (FAO 2015). In order to enhance productivity, improving salt tolerance of crop plants has the potential to make marginal areas agriculturally productive (Foolad 2007, Karan and Subudhi 2012). To achieve this goal, it is crucial to understand the physiological, biochemical and molecular mechanisms evolved by plants to cope with salt stress.

Soil salinization inhibits water uptake by the plants, causes ionic imbalance leading to ionic toxicity and osmotic stress (Munns and Tester 2008). To withstand salt stress, plants accumulate compatible solutes such as proline, which decreases the cytoplasmic osmotic potential, facilitating water absorption, and scavenges reactive oxygen species (ROS) molecules (Qureshi *et al.* 2013; Pottosin *et al.* 2014).

Multiple signalling pathways lead to the expression of genes that in turn allow the activation of the proteins that determine plant phenotype under salt stress (Marco *et al.* 2015). Data on signalling pathways have increased in recent years. Analysis of this data will not only elucidate the function and regulation of complex plant responses to salt stress but also the identification of genes whose function is unknown and which may have important roles in salt tolerance. These downstream signalling pathways comprise several active components including second messengers, phytohormones and phosphoprotein cascades. The Ca^2+ ^ is a second messenger in signalling network coupling the perception of a stressful environment to a significant plant adaptability (Tuteja and Mahajan 2007; Marco *et al.* 2015). Ca^2+ ^ acts at the crossroads of various signalling pathways (Gill and Tuteja 2010; Rany *et al.* 2016). High-salinity stress initiates the calcium signalling network (Tuteja 2009), inducing membrane depolarization, and may activate sensitive Ca^2+ ^ channels to generate a Ca^2+ ^ signature (Tester and Davenport 2003; Zhu 2003). Increases in Ca^2+ ^ concentrations and stimulus-induced enhancement in Ca^2+ ^ sensitivity (Young *et al.* 2006) function as an effective signal which modulates calcium-binding proteins thus transmitting signals in signal transduction pathways (Uozumi and Schroeder 2010).

Phytohormones such as abscisic acid (ABA), salicylic acid (SA), ethylene (ET) and jasmonic acid (JA) activate pathways that may act independently or synergistically with others triggered by stress (Marco *et al.* 2015). Protein kinases and phosphatases play a fundamental role in the coordination of the activity of many known signalling pathways (Marco *et al.* 2015). Transcriptome studies reveal that genes induced by these signalling cascades triggered by salt stress can be divided into two categories depending on the features of their products (Bohnert *et al.* 2001; Fowler and Thomashow 2002; Seki *et al.* 2002). The first, composed of functional proteins such as membrane proteins, protects cells against stress effects by restoring cellular homeostasis.

Ion channels in plant cells play crucial functions in adapting and overcoming salt stress (Uozumi and Schroeder 2010). Cation transporters as *HKT* and *LeNHX* enhance salt tolerance by regulation internal concentrations of Na^+ ^ in tissues. The expression level of HKT1-like transporters has been reported to be directly related to salt tolerance and Na^+^-specific tissue distribution depending to the plant source. *HKT1;1* and *HKT1;2* are two tomato Na^+^-^ ^selective transporters that contribute to Na^+ ^ and K^+ ^ homeostasis (Hauser and Horie 2010; Pardo and Rubio 2011). Salt tolerance is achieved by retrieval of Na^+ ^ from the xylem vessels to xylem parenchyma cells, promoting vacuolar accumulation and thus protecting photosynthetic leaf tissues from the adverse effect of Na^+^(Davenport *et al.* 2007; Plett *et al.* 2010, Xue *et al.* 2011; Munns *et al.* 2012). Several studies reported that HKT-I like transporters are associated with QTL on chromosome 7 in two populations of F(8) lines, derived from a salt sensitive genotype of *Solanum* *lycopersicum cv. Cerasiforme*, as female parent and two salt tolerant lines, as male parents, from *Solanum* *pimpinellifolium* and *Solanum* *cheesmaniae*. HKT-I like transporters seem to be involved in Na^+ ^ and K^+ ^ homeostasis in aerial parts of the plant (Ren *et al.* 2005; Villalta *et al.* 2008). *NHX* (Na^+^/H ^+^  Antiporters) and *HKT* (*Histidine Kinase Transporter*) genes, encoding K^+ ^ transporters and channels, have been implicated in multiple biological responses in various plant species (Gupta and Huang 2014). The two *LeNHX1* and *LeNHX2* isoforms localized in the tonoplast are essential for active K^+ ^ uptake, for stomatal function and for turgor regulation (Barragán *et al.* 2012) while *LeNHX3* and *LeNHX4* isoforms are involved in Na^+^, K^+^, and H^+ ^ homeostasis (Gàlvez *et al.* 2012). The *HKT* family improves salt tolerance by regulating ion transportation (Gupta and Huang 2014). In tomato, *HKT1;1* and *HKT1;2* are responsible for the major QTL involved in Na^+ ^ and K^+ ^ homeostasis (Asins *et al.* 2012). In *Arabidopsis*, *HKT* transporters protect the plant from the adverse effects of salinity by preventing excess Na^+ ^ accumulation in leaves. Experiments carried out on rice by Schroeder *et al.* (2013) suggest that *HKT* class I transporters remove excess Na^+ ^ from xylem, protecting the photosynthetic leaf tissues from the toxic effect of Na^+^. This first category also includes biosynthetic enzymes for metabolites acting in osmotic adjustment or protection as well as ROS detoxification enzymes.

High salinity has been reported to induce ROS formation and accumulation in plant cells (Chawla *et al.* 2013). Oxidative stress defenses occur through enzymatic antioxidant mechanism including catalase (CAT), superoxide dismutase (SOD), peroxidase (POX) and enzymes of the ascorbate-glutathione cycle as ascorbate peroxydase (APX), monodehydroascorbate dehydrogenase (MDHAR), dehydroascorbate reductase (DHAR) (Foyer and Noctor 2011; Chawla *et al.* 2013) and non-enzymatic antioxidants as phenolics, flavonoids (Munné-Bosch 2005; Gupta and Huang 2014; Rakhmankulova *et al.* 2015; Talbi *et al.* 2015). CAT is involved in scavenging of H_2_O_2_ during salt stress and other abiotic stress conditions (Willekens *et al.* 1997) and is considered as a major enzyme detoxifying H_2_O_2_ in tomato fruits (Murshed *et al.* 2014). Although APX performs the same general function as catalase, it catalyzes removal of H_2_O_2_ by using ascorbate as a reductant. APX is a family of isozymes widely involved in regulation of intracellular level of H_2_O_2_ in higher plants (Van Breusegem *et al.* 2001; Shigeoka *et al.* 2002). GPOX enzymes protect cells against oxidative damage generated by ROS. They catalyze the reduction of H_2_O_2_ or organic hydroperoxides to H_2_O or alcohols. The second category comprises a series of regulatory proteins (transcription factors, protein kinases) involved in the regulation of the signalling cascade that controls the expression of additional genes whose products could belong, in turn, to either of the two groups (Agarwal *et al.* 2006; Shinozaki and Yamaguchi-Shinozaki 2007).

The main stress-related transcription factors include members of *APETALA2*/*Ethylene Responsive Factor* (AP2/ERF), basic helix-loop-helix (bHLH), and basic leucine zipper (bZIP) proteins, the homeodomain-leucine zipper (HD-Zip), myelocytomatosis (MYC), myeloblastosis (MYB) and NAC families and members of the *WRKY* family (Lindemose *et al.* 2013). Previous studies revealed a significant induction of 18 different tomato *SlWRKY* genes under conditions of salt, drought or pathogen challenge, implying that they are regulators of plant responses to various biotic and abiotic stresses (Huang *et al.* 2012). Involvement of *WRKY* factors in plant salt adaptation was shown for *WRKY15*; *18*; *20*; *25*; *33*; *39*; *40*; *45*; *60* and *WRKY82* which increased salt tolerance in many plant species (Bakshi and Oelmüller 2014; Huang *et al.* 2012; Jiang and Deyholos 2009; Liu *et al.* 2011; Peng *et al.* 2012; Sun *et al.* 2014). These transcription factors are well interconnected with other complex signalling pathways corresponding to cell homeostasis, photosynthesis, oxidative pathway and enzyme activity (Baniwal *et al.* 2007; Eulgem and Somssich 2007). In *Arabidopsis, AtWRKY25* and *AtWRKY33* transcription level was increased by drought or NaCl treatment (Chen *et al.* 2012). Sharma *et al.* (2010) reported the induction of *ERF* family genes in response to various stress treatments (salt, cold, heat, dehydration, mechanical stress, oxidative stress, and submergence stress) suggesting a crosstalk between different stress-signalling pathways. All these elements interact with each other, forming a complex network which finally results in the modification of target proteins that may have enzymatic or structural function, leading to cellular responses at the physiological, biochemical and molecular levels.

Because of the extensive salinization of Tunisian soils, considerable efforts are being invested to improve salt tolerance in tomato. Achieving this goal depends on elucidation of mechanisms by which tomato plants are able to perceive stress and to activate appropriate cellular responses. In the present study, we explore the behaviour of tomato genotypes subjected to saline treatment to discover whether genotypic differences in response to salt stress exist. We explored the modulation of some physiological traits involved in the response to salt stress, mainly Na^+^, K^+^, Ca^2+^, and proline content, as well as expression of the antioxidant enzymes APX, CAT and GPOX. We also investigated the expression of a panel of genes (*WRKY*, *LeNHX*, *HKT* and *ERF*) involved in ion accumulation, encoding ion transporters or channels. Analyses were performed in leaves and roots at early (0, 6 and 12 h) and late (7 days) stages of the saline treatment. Our findings will help identifying potential candidate genes for local tomato genetic improvement to salt stress in Tunisia.

## Methods

### Plant material

Twenty tomato genotypes commonly cultivated by Tunisian growers were used in this investigation. They correspond to three TYLCV-tolerant lines (San Miguel, Ilanero and Romelia; Scott *et al.* 1995; Vidavsky and Czosnek 1998; Mejía *et al.* 2002) and 17 local Tunisian genotypes. Seeds were surface sterilized with 0.5 % NaCl solution, rinsed with water and incubated in Petri dishes on moist sterile filter paper at 27 °C in darkness until emergence of the radicle. Two days later, tomato seedlings were transferred into hydroponic tanks, each containing 10 L of half-strength modified Hoagland solution (Epstein 1972). The hydroponic solutions were vigorously aerated and renewed every 2 days during the growing period. The experiment was carried out with three replicates [**see Supporting Information—Figure S1**]. Plants were grown in an environmentally controlled chamber at 25 °C/18 °C, day/night and a 16-h light/8-h dark cycle with 40–50 % relative humidity (Asins *et al.* 2012).

### Evaluation of salt tolerance

Plants with four fully developed true leaves were individually transferred into plastic pots (30 cm of diameter) containing a mixture of peat and sand then irrigated with one-half Hoagland solution supplemented with 150 mM NaCl (15 dS/m, pH 7.5). Salt treatment was initiated with 50 mM of NaCl solution (6 dS/m), increased to 100 mM (12 dS/m) on day two and finally to 150 mM (15 dS/m) on day three. We used three biological replicates for each of the 20 varieties. Each replicate consisted of a pool of 10 plants. A set of three plants for each genotype was grown in non-saline conditions and watered with the nutrient solution. Three weeks later, salt-treated plants were evaluated for salt tolerance, based on their visual phenotypes compared to control plants. Plants were rated for severity of salt susceptibility by on a 1–5 scale (Dasgan *et al.* 2002) [**see Supporting Information—Figure S2**].

Ten plants of each genotype were tested at 0 h, 6 h, 12 h and 7 days post-saline treatment. Fresh Root and leaf tissues were harvested, rinsed with demineralized water and weighted for immediate use. Half of the plants were dried in a forced air oven at 70 ºC to determine the dry weight (DW) [**see Supporting Information—Figure S3**]. The remaining plants were immediately stored at −80 ºC.

### RNA isolation and quantitative real-time PCR analysis

Total RNA was isolated from root and leaf tissues using the TRIzol^®^ LS Reagent (Trizol RNA stabilization solution, Invitrogen; Life Technologies, Carlsbad, CA, USA) according to the manufacturer’s instructions. RNA was quantified by ND-1000 spectrophotometer (Nanodrop Technologies, USA). First-strand cDNA was synthesized from 2 µg of total RNA with oligo(dT) and MMLV reverse transcriptase (200 U/μl, Invitrogen) according to the manufacturer’s instructions. ABI A Prism 7000 sequence detection system (Applied Biosystems, USA) was used for quantitative real-time PCR (qPCR) under the following cycle conditions: 10 min at 95 °C followed by 40 cycles of 15 s at 95 °C, 1 min at 60 °C. The *Actin* tomato gene (*ACT*) was used as internal reference gene (Lovdal and Lillo 2009 [**see Supporting Information—Figures S3 and S4**]. Genes and their corresponding primers are shown in Table 1. PCR reactions were carried out in 96-well optical reaction plates (Applied Biosystems, USA). Reaction included 50 ng of cDNA sample as a template, 400nM forward and reverse primers, and Igreen qPCR master Mix-Rox (BIOMATIK, USA). Relative quantification was performed by applying the 2^−^^ΔΔCt^ method (Livak and Schmittgen 2001).

**Table 1.** List of primers used for qPCR analysis.

**Genes names**	**Primers**	**References**
SIWRKY 8	F: TAATTCTGCCGGAAAGCCTC R: ATGCTTATTGCCGGTACTCGA	Huang *et al.* (2012)
SIWRKY 31	F: ACAACCTATGAAGGGAAGCACA R: AGGGTGCTCCCATTTCAGAC	
SIWRKY 39	F: GCGGTAATGCCAAGACAAAC R: TCAGTTCCTGGTGATTTACGC	
SIERF 9	F: TGGAAGGAGTATAATGAGAAACTA GAC AA R: CCT TCTTTGAACCTTTAGCAGGAA	Sharma *et al.* (2010)
SIERF 16	F: GCGAATAATACAGAACCCGAACTT R: TGAGGAAGAAGAAAGATCCGAATT	
SIERF 80	F: TTTCAATCATGGTTGCTGCTTT R: AAGGGCGGCGACATACC	
LeNHX1	F: GACAGTCCTGGAAAATCT R: GGTTATCAGCCCAAACACC	Gálvez *et al.* (2012)
LeNHX3	F: CTCAAGAGTCACCACCAAGCA R: CCAACCAAAACAAGACCCAACA	
LeNHX4	F: TGGTGGGCAGGTTTGATGAGAG R: TGTGGTGGCAGCAGGAGACTTA	
HKT1.1	F: TCTAGCCCAAGAAACTCAAAT R: CTAATGTTACAACTCCAAGGAATT	Asins *et al.* (2012)
HKT1.2	F: TGAGCTAGGGAATGTAATAAACG R: AGAGAGAAACTAACGATGAACC	
*ACTIN*	F: GAAATAGCATAAGATGGCAGACG R: ATACCCACCATCACACCAGTAT	Lovdal and Lillo (2009)

### Na^+^, K^+ ^ and Ca^2+ ^ measurements

Dried plant materials consisting to roots and leaves were digested with HNO_3_/HClO_4_ solution (2:1). Na^+ ^ and K^+ ^ ions were quantified using a flame photometer (Jenway Model PEP7, USA). Ca^2+ ^ concentrations were determined by atomic absorption spectrometry (Perkin-Elmer 5 5500, Waltham, MA, USA).

### Proline concentration

Proline content in root and leaf tissues was measured via reaction with ninhydrin (Bates *et al.* 1973). For colorimetric determinations, a solution of proline, ninhydrin acid and glacial acetic acid (1:1:1) was incubated at 90 ºC for 1 h. Then, the reaction was cooled in an iced bath. The chromophore was extracted using 2 ml of toluene and its absorbance at 520 nm was determined by a BioMate spectrophotometer (ThermoSpectronic, USA).

### Antioxidant enzyme activity measurements

One gram of either fresh leaf or root material was weighted individually and immediately homogenized in 5 ml of 50 mM K–phosphate buffer (pH 7.0), brought to 5 mM Na–ascorbate and 0.2 mM EDTA by the addition of concentrated stocks. The homogenate was centrifuged at 10 000*g* for 15 min at 4 °C. The resulting supernatant was used for enzyme assays. The extraction was carried out at 4 °C. Proteins were quantified according to Bradford (1976) using albumin bovine serum as a standard.

Activity of CAT was determined by monitoring the disappearance of H_2_O_2_ at 240 nm (extinction coefficient of 0.036 mM ^−^ ^1^ cm ^−^ ^1^) (Aebi 1984). One unit of activity was defined as the amount of enzyme required to decompose 1 µmol H_2_O_2_ per min at 25 °C. Activity of GPOX was measured by monitoring the increase in absorbance at 470 nm (ε = 26.6 mM ^−^ ^1^ cm ^−^ ^1^) during polymerization of guaiacol (Fielding and Hall 1978). One unit of activity was defined as the amount of enzyme producing 1 µmol of tetraguaiacol per min at 25 °C. Activity of APX was determined based on the decrease in absorbance at 290 nm (absorbance coefficient 2.8 mM^−^^1^ cm^−1^) as ascorbate was oxidized according to Nakano and Asada (1981). One unit of enzyme was defined as the amount necessary to decompose 1 µmol of ascorbate per min at 25 °C.

### Statistical analysis

Data were analyzed using two-way ANOVAs with times and varieties as the two predictor variables. Differences at Tukey’s test HSD_p = 0.05_ were considered statistically significant. Analyses were performed using GraphPad Software (version 6.0, CA, USA). A heat map and a signal correlation were performed to visualize the correlation of the expression of candidate genes during salt treatment based on Pearson’s correlation. Only the comparisons with *P* < 0.05 were regarded as showing differential expression. The neutral/middle expression was set as the median of all the Δ*C*_t_ values from tested varieties, red colour was used to indicate an increase with a Δ*C*_t_ value below the median and the green indicated a decrease with Δ*C*_t_ above the median. The Δ*C*_t_ set of each considered variety is plotted on a scatter graph where the two axes are the Pearson correlation coefficients against two different query Δ*C*_t_ sets. Analyses were performed with DataAssist™ v3.0 Software (Applied Biosystems, USA).

## Results

### Evaluation of tomato genotypes under salt stress

**Figure 1.**
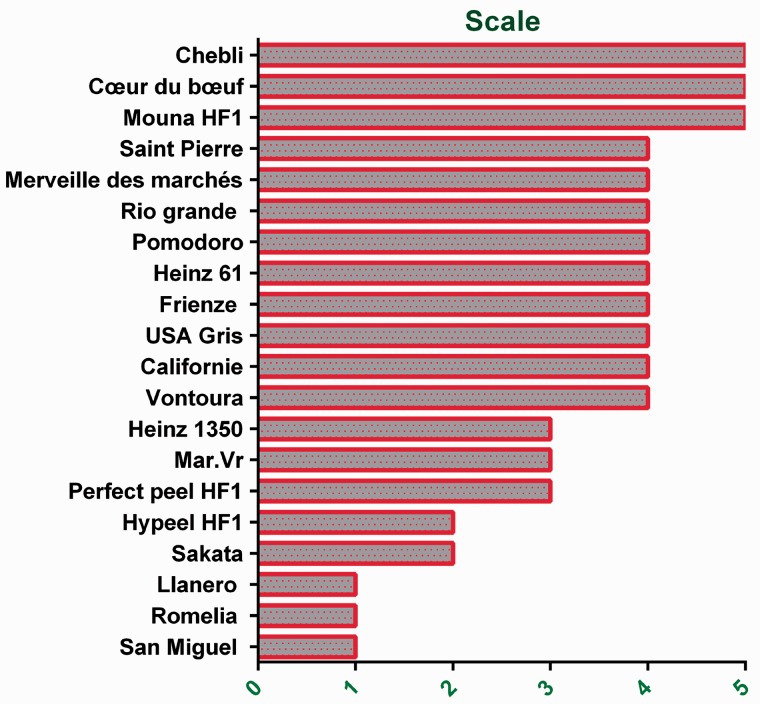
Clustering of Tunisian tomato genotypes based on their phenotypes according to Dasgan' scale classes (Dasgan *et al.* 2002).

**Figure 2.**
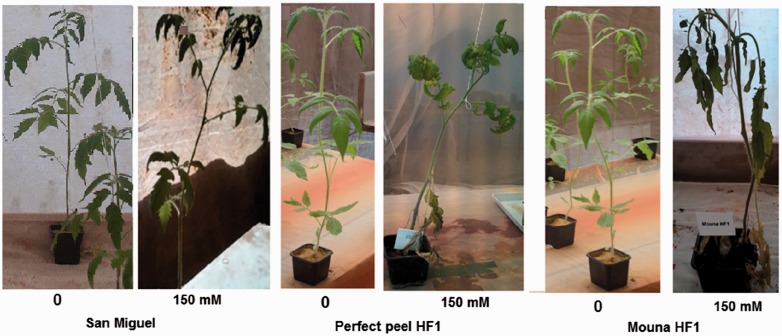
Visual appearance of the three selected genotypes growing in saline and non-saline conditions.

Based on symptoms and visual phenotypes, tomato varieties were screened for their response to the salt stress treatment according to Dasgan's (Dasgan *et al.* 2002) 1–5 scale classes. Three of the evaluated varieties (San Miguel, Ilanero and Romelia) showed no visual symptoms of dehydration and were clustered to the Class 1. Remaining genotypes were assigned to scale-classes as follow: 10 % (Sakata and Hypeel HF1) to Class 2, 15 % (Perfect peel HF1, Mar.Brand Heinz 1350) to class 3, 45 % (Ventura, California, USA gris, Frienze, Heinz 61, Pomodoro, Rio grande, Merveille des marchés and Saint Pierre) to Class 4 % and 15 % (Mouna HF1, Coeur du boeuf and Chebli) to Class 5 (Fig. 1). Salt treatment seems not to affect the maintenance of the growth of Class 1 genotypes while leading to reduced stem growth of those belonging to Classes 4 and 5. Subsequent analyses focused on three contrasting varieties. San Miguel represented the tolerant scale-Class 1 without any symptoms of salt damage; Perfect peel HF1 represented the mildly tolerant scale-Class 3 with curly and moderate dry leaves and the sensitive Mouna HF1 belonged to the scale-Class 5 showing all leaves with drying damage (Fig. 2).

### Na^+^, K^+ ^ and Ca^2+ ^ contents

**Figure 3.**
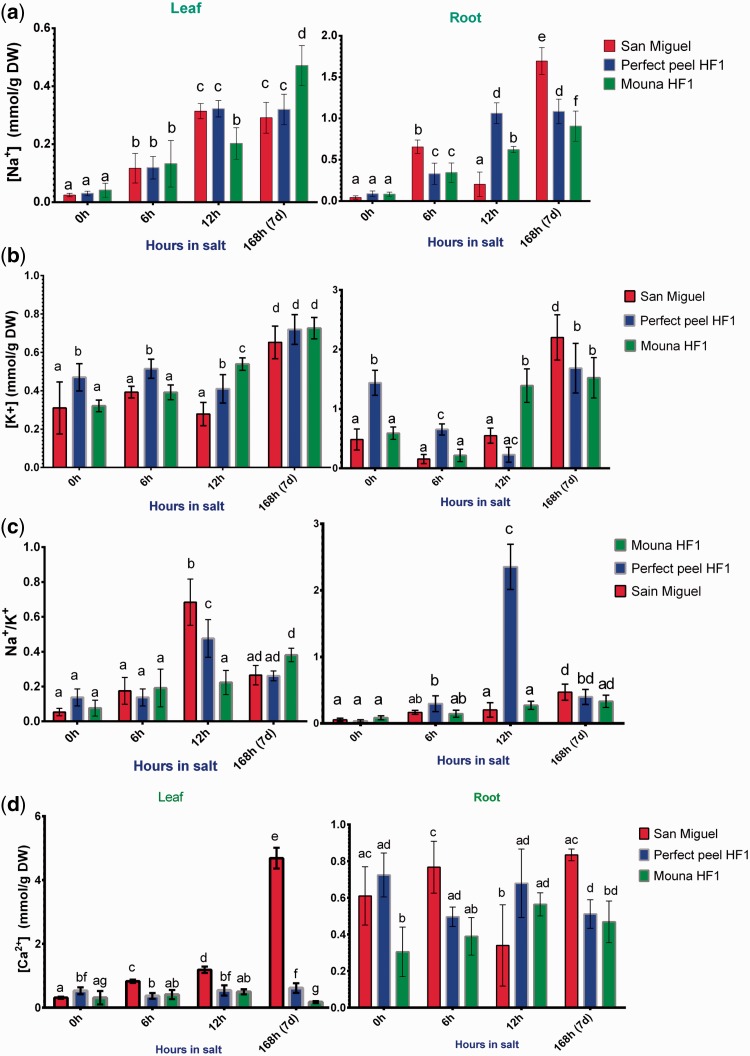
Ion content in leaf and root tissues within San Miguel, Perfect peel HF1 and Mouna HF1 genotypes during 6 h, 12 h and 7 days post-NaCl treatment (15 dS/m, pH 7.5). (a) Na^+^, (b) K^+^, (c) Na^+^/K^+^ and (d) Ca^2+^. Values are expressed in mmole/g DW. The data reported are the mean ± SE of five biological replicas. Bars with different letters within each panel are significantly different at *P *> 0.05 according to Tukey's test.

The distribution of Na^+^, K^+ ^ and the ratio Na^+^/K^+ ^ as well as Ca^2+ ^ in both leaves and roots were analyzed at 6 h, 12 h and 7 days post-stress application. Depending on the genotype, the increase in Na+  concentration followed a constant pattern in leaves (*F*_2, 8 _=_ _1.547, *P* = 0.2705) and varied significantly in roots (*F*_2, 8 _=_ _9.190, *P* = 0.0085). Na+  concentration also varied significantly according to the salt stress stage treatment in both tissues (Leaves: *F*_3, 12 _=_ _104.8, *P* < 0.0001; Roots: *F*_3, 12 _=_ _172.4, *P* < 0.0001). We also detected a significant interaction between genotypes and stages of stress treatment (*F*_6, 24 _=_ _11.75, *P* < 0.0001) and (*F*_6, 24 _=_ _67.87, *P* < 0.0001) in leaves and roots, respectively. In early stage of stress treatment (6 h), the three tested genotypes displayed similar Na^+ ^ concentrations in their leaves. By contrast, at 7 days post-treatment, the sensitive Mouna HF1 showed the highest Na^+ ^ concentration. Regardless of genotype, Na^+ ^ preferentially accumulated in roots rather than in leaves (Fig. 3A). Results showed the tolerant San Miguel accumulating the highest Na^+ ^ level in roots at the latest stage of salt treatment. Results from two-way ANOVA indicated that stages of treatment (leaves: *F*_3, 12 _=_ _77.31, *P* < 0.0001; roots: *F*_3, 12 _=_ _108.0, *P* < 0.0001) and genotypes (leaves: *F*_2, 8 _=_ _17.70, *P* = 0.0012; roots: *F*_2, 8 _=_ _5.674, *P* = 0.0292) had a significant overall effect on K^+ ^ concentration in tissues. In addition, we detected significant stages × genotypes interaction terms for both tissues (leaves: *F*_6, 24 _=_ _5.176, *P *= 0.0015; roots: *F*_6, 24 _=_ _20.67, *P* < 0.0001).K^+ ^ showed the same levels in leaves in all three genotypes (after 7 days of treatment). In roots, San Miguel and Mouna HF1 displayed similar K^+ ^ concentrations at early (6 h) stage of treatment whereas K^+ ^ content concentration increased within San Miguel at the latest stage (7 days) (Fig. 3B). As it appears to be a key determinant of salt tolerance, the Na^+^/K^+ ^ ratio was calculated Na+/K+  is statistically significant between genotypes (leaves: *F*_2, 8 _=_ _5.864, *P* = 0.0270; roots: *F*_2, 8 _=_ _341.2, *P* < 0.0001) and between stages of salt stress (leaves: *F*_3, 12 _=_ _145.8, *P* < 0.0001; roots: *F*_3, 12 _=_ _94.47, *P* < 0.0001). In addition, we detected significant stage × genotype interaction terms for both tissues (leaves: *F*_6, 24 _=_ _12.97, *P* < 0.0001; roots: *F*_6, 24 _=_ _120.7, *P* < 0.0001). In leaves, San Miguel and Perfect peel HF1 exhibited the highest leaf Na^+^/K^+ ^ ratio at 12 h post salt-treatment. All genotypes showed similar Na^+^/K^+ ^ ratio in both leaves and roots at the latest stage of treatment (Fig. 3C).

In order to initiate defence–response mechanisms during saline stress, plants first need to perceive the stress, then activate the whole signalling cascade, starting with an increase of Ca^2+ ^ concentration. For this reason, Ca^2+ ^ concentration was also determined. Depending on the genotype, the increase in Ca^2+ ^ concentration followed a different pattern in leaves (*F*_2, 8 _=_ _520.4, *P* < 0.0001) and roots (*F*_2, 8 _=_ _25.15, *P* = 0.0004). Results revealed that stages of salt treatment had significant effect on Ca^2+ ^ concentration in leaves (*F*_3, 12 _=_ _214.5, *P* < 0.0001) but not in roots (*F*_3, 12 _=_ _0.9336, *P* = 0.4546). We also detected a significant interaction between these variables in tissues (leaves: *F*_6, 24 _=_ _403.4, *P* < 0.0001; roots: *F*_6, 24 _=_ _8.902, *P* < 0.0001). The highest Ca^2+ ^ concentration was recorded in San Miguel (the tolerant genotype) in both leaf and root tissues, at the latest stage of the treatment. By contrast, Perfect peel HF1 and Mouna HF1 genotypes showed reduced Ca^2+ ^ in leaves during first stage of salt stress. At 7 days post-salt imposition, both displayed the lowest lower Ca^2+ ^ concentrations in leaves and roots (Fig. 3D).

### Effects of saline stress on proline accumulation

**Figure 4.**
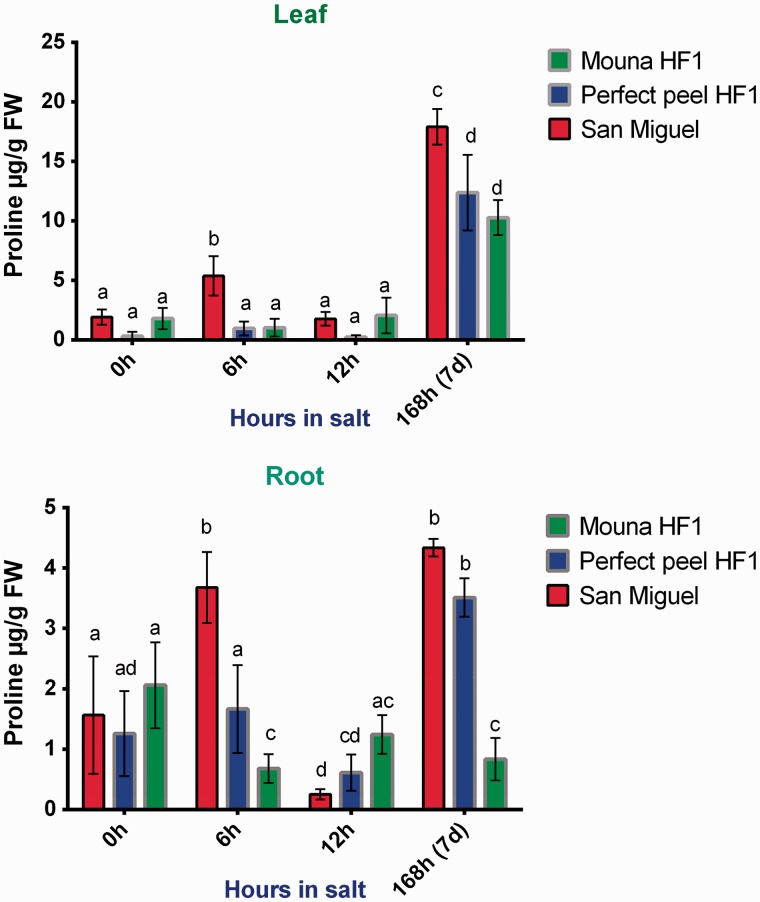
Proline accumulation in leaf and root tissues within San Miguel, Perfect peel HF1 and Mouna HF1 genotypes during 6 h, 12 h and 7 days post NaCl treatment (15 dS/m, pH 7.5). Data expressed as µg/g of fresh weight are the mean ± SE of 3 biological replicas. Bars with different letters within each panel are significantly different at *P *> 0.05 according to Tukey's test.

To combat osmotic stress imposed by high salinity, plants need to synthesize compatible organic solutes such as proline in the cytosol. Accumulation of proline was determined and seems to be linked to the scale class. Results from two-way ANOVA and Tukey's test indicated that the stage of salt treatment had a significant overall effect on proline concentration (leaves: *F*_3, 12 _=_ _260.2, *P* < 0.0001; roots: *F*_3, 12 _=_ _44.51, *P* < 0.0001). Similarly, results showed proline concentration varied significantly between genotypes in both tissues (leaves: *F*_2, 8 _=_ _48.22, *P* < 0.0001; roots: *F*_2, 8 _=_ _37.69, *P* < 0.0001). In addition, we detected significant interaction terms between genotypes and stages of treatment for leaves (*F*_6, 24 _=_ _8.949, *P* < 0.0001) and roots (*F*_6, 24 _=_ _23.20, *P* < 0.0001). In leaves, the tolerant San Miguel genotype showed a negligible content of proline during first stages of treatment with a peak observed at 6 h. However, proline concentration increased significantly at the late stage of the treatment, reaching 17 µg/g FW. Perfect Peel HF1 and Mouna HF1 genotypes displayed lower proline concentration at 7 days post-treatment reaching 13 µg/g FW and 10 μg/g FW, respectively (Fig. 4A). In roots, proline amount reached 4.33 µg/g FW, 3.50 µg/g FW and 0.82 µg/g FW in San Miguel, Perfect peel HF1 and Mouna HF1, respectively, at 7 days post-salt treatment (Fig. 4B). It is worth noting that the tolerant San Miguel genotype displayed similar proline concentration to Perfect peel HF1 at the latest stage of the treatment. Results indicate that proline is more abundant in leaves than roots within all the stressed genotypes especially at the end of the treatment.

### Antioxidant enzyme activities

**Figure 5.**
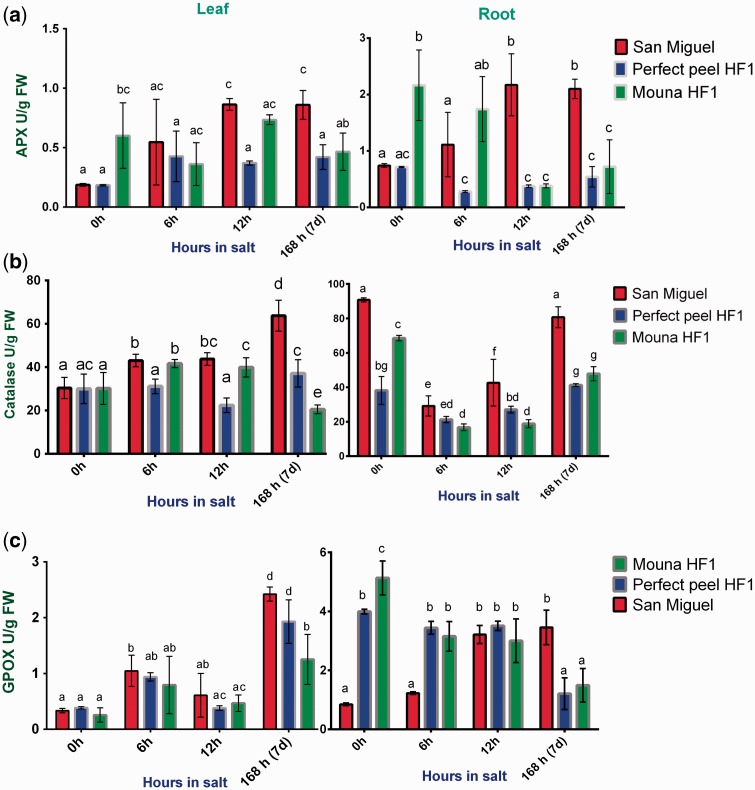
Antioxidative enzyme activities in leaf and root tissues within San Miguel, Perfect peel HF1 and Mouna HF1 genotypes during 6 h, 12 h and 7 days post-NaCl treatment**.** (a) APX, (b) Cat and (c) GPOX. Values are the mean ± SE of 3 biological replicas. Bars with different letters within each panel are significantly different at *P *>* *0.05 according to Tukey's test.

To minimize the deleterious effects of ROS, plant cells suppress the buildup of harmful intracellular ROS concentrations. This is achieved by the action of the antioxidative defence systems as enzymatic ROS scavengers including APX, CAT and GPOX. In order to get further insight into the effect of salt stress on oxidative stress parameters, APX, CAT and GPOX activities were measured. Two way ANOVA followed by Tukey’s multiple comparisons test indicated that variation in APX activity is statistically significant between genotypes in roots (*F*_2, 4 _=_ _57.23, *P* = 0.0011) but not in leaves (*F*_2, 4 _=_ _6.382, *P* = 0.0569). By contrast, APX activity is significantly different between stages of stress treatment in leaves (*F*_3, 6 _=_ _6.252, *P* = 0.0282) but not in roots (*F*_3, 6 _=_ _0.3765, *P* = 0.7736). When considering interaction between genotypes and stages of stress treatment, results indicated that variation is statistically significant (leaves: *F*_6, 12 _=_ _3.976, *P *= 0.0201; roots: *F*_6, 12 _=_ _14.90, *P* < 0.0001). In both leaf and root tissues, APX activity gradually increased during all stages of the stress treatment within the San Miguel tolerant plant, whereas it was reduced with the remaining plants, especially in roots (Fig. 5A). CAT activity was not associated with significant changes at any stage of salt treatment in leaves (*F*_3, 6 _=_ _4.487, *P* = 0.0562) but not in roots (*F*_3, 6 _=_ _194.9, *P* <0.0001). CAT activity increased significantly among genotypes (leaves: *F*_2, 4 _=_ _25.48, *P* = 0.0053; roots: *F*_2, 4 _=_ _91.06, *P* = 0.0005). We also detected a significant interaction between genotypes and stages of stress treatment (leaves: *F*_6, 12 _=_ _29.81, *P* < 0.0001; roots: *F*_6, 12 _=_ _14.62, *P* < 0.0001). Leaf CAT activity displayed a significant increase within San Miguel at the latest stage post-salt treatment (Fig. 5B). Concerning GPOX activity, statistical results showed significant changes in both tissues either between genotypes (leaves: *F*_2,4 _=_ _13.06, *P* = 0.0176; roots: *F*_2, 4 _=_ _35.46, *P* = 0.0029) or between stages of salt treatment (leaves: *F*_3, 6 _=_ _31.75, *P* = 0.0004; roots: *F*_3, 6 _=_ _7.946, *P* = 0.0164). The interaction between these two variables is also significant (leaves: *F*_6, 12 _=_ _3.293, *P* = 0.0374; roots: *F*_6, 12 _=_ _52.82, *P* < 0.0001). GPOX leaf activity displayed the highest value within San Miguel and Perfect Peel HF1 genotypes at the end of the treatment (Fig. 5C). Otherwise, GPOX root activity showed a similar pattern within Perfect Peel HF1 and Mouna HF1 during the first and the last stage of treatment increasing within San Miguel at 7 days post-stress.

### Analysis of differentially expressed (*WRKY*, *ERF*, *LeNHX* and *HKT*) genes by qRT-PCR

The expression profiles of tomato genes were analyzed in both leaf and root tissue. Three *ERF* family genes (*ERF9*, *16* and *80*), three *WRKY* family genes (*WRKY8*, *31* and *39*), 2 *HKT* class I gene transporters (*HKT1;1* and *1;2*) and three *LeNHX* genes (*LeNHX1*, *3* and *4*) were selected and subjected to a qRT-PCR analysis for samples corresponding to the first stage (0 h, 6 h and 12 h) and a last stage (7 days) of the stress imposition.

**Figure 6.**
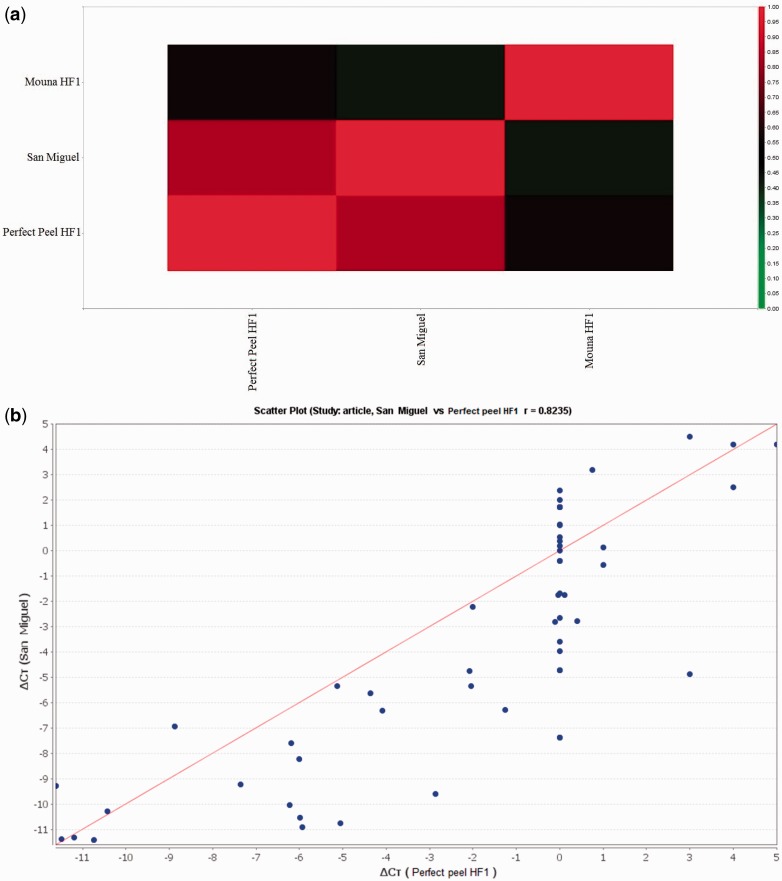
(A) Signal correlation of San Miguel F1, Perfect peel HF1 and Mouna HF1 genotypes. The colour scale represents relative expression levels with red as increased transcript abundance and green as decreased transcript abundance. (B), (C) and (D) represent the scatter plot for global expression between San Miguel, Perfect Peel HF1and Mouna HF1, repectively. The Pearson correlation coefficient “*r*” is shown.

Heat maps of transcript expression were constructed [**see Supporting Information—Figure S5**] and genotype correlation analyses conducted. The correlation signal showed that the gene expression profile of San Miguel was very similar to that of Perfect peel HF1. In contrast, the expression profiles of San Miguel and Perfect peel HF1 were quite distinct from that of Mouna HF1 (Fig. 6A). In order to compare mRNA expression profiles in leaf and root tissues of the examined genotypes, correlation coefficients were calculated and showed in scatter plots. Analysis revealed a high-correlation coefficient (*r* = 0.82) between San Miguel and Perfect peel HF1, indicating that these two varieties are highly correlated with regard to the selected genes (Fig. 6B). By contrast, comparison of gene expression profiles pointed to a low correlation between San Miguel vs. Mouna HF1 and Perfect peel HF1 vs. Mouna HF1 (*r* = 0.42 and *r* = 0.56, respectively; Fig. 6C and D).

### *WRKY* genes

**Figure 7.**
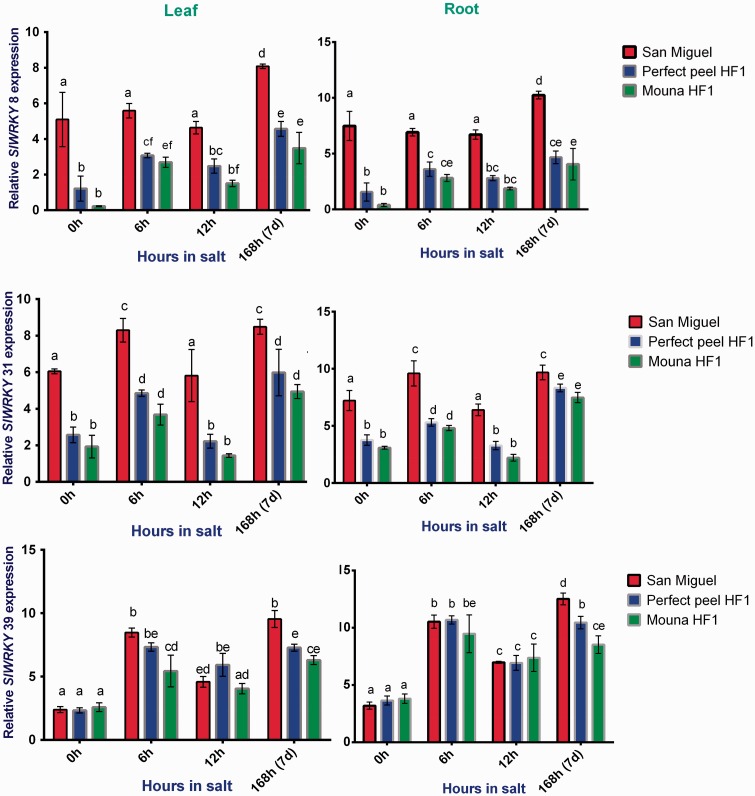
Relative gene expression of tomato *WRKY8*, *WRKY31* and *WRKY39* in response to salt stress in leaves and roots of San Miguel, Perfect peel HF1 and Mouna HF1 genotypes. Total RNA was purified from tissues of tomato plants treated with 150 mM NaCl for 0 h, 6 h, 12 h and 7 days. Transcript level was analyzed by qRT-PCR using primers indicated in Table 1. Tomato *Actin* gene was used as reference gene. Error bars show the standard error between three replicates performed. Bars with different letters within each panel are significantly different at *P *> 0.05 according to Tukey's test.

In response to salt stress, *WRKY* genes showed up-regulated expression and displayed distinct patterns. Two-way ANOVA followed by Tukey’s multiple comparisons test indicated that relative *SIWRKY*8 and *31* expression is statistically significant between genotypes (leaves: *F*_2, 4 _=_ _316.5, *P* < 0.0001; roots: *F*_2, 4 _=_ _195.7, *P*= 0.0001) and (leaves: *F*_2, 4 _=_ _228.4, *P* < 0.0001; roots: *F*_2, 4 _=_ _399.7, *P* < 0.0001), respectively. *SIWRKY39* expression was significantly different in leaves (*F 2, 4 *=* *44.80, *P* = 0.0018) but not in roots (*F_2, 4 _*=_* *_6.077, *P* = 0.0613). Expression of *SIWRKY8*, *31* and *39* varied significantly between stages of salt stress (leaves: *F*_3, 6 _=_ _53.74, *P* < 0.0001; roots: *F*_3, 6 _=_ _69.92, *P*< 0.0001), (leaves: *F*_3, 6 _=_ _105.2, *P* < 0.0001; roots: *F*_3, 6 _=_ _245.8, *P* < 0.0001) and (leaves: *F*_3, 6 _=_ _198.0, *P* < 0.0001; roots: *F*_3, 6 _=_ _223.3, *P* < 0.0001). Results also indicated that interaction between genotypes and stages of treatment are not statistically different for *SIWRKY8* (leaves: *F*_6, 12 _=_ _2.351, *P* = 0.0978; roots: *F*_6,12 _=_ _2.821, *P* = 0.0596) and for *SIWRKY31* in leaves (*F*_6, 12 _=_ _0.6028, *P* = 0.7238). By cons, such interaction is supported statistically for *SIWRKY31* in roots (*F*_6, 12 _=_ _4.068, *P* = 0.0185) and for *SIWRKY39* in both tissues (leaves: *F*_6, 12 _=_ _7.349, *P* = 0.0018; roots: *F*_6, 12 _=_ _6.680, *P* = 0.0027). Within San Miguel, transcription of *SlWRKY8* (Group II-d) was enhanced with saline treatment, particularly at 7 days whereas that of *SlWRKY31* (Group I) showed similar expression pattern in both leaves and roots *SlWRKY39* gene was expressed similarly between San Miguel and Perfect Peel HF1 genotypes during first stage of the treatment but showed increased expression relative to the tolerant one at the end of the treatment (Fig. 7).

### *ERF* genes

**Figure 8.**
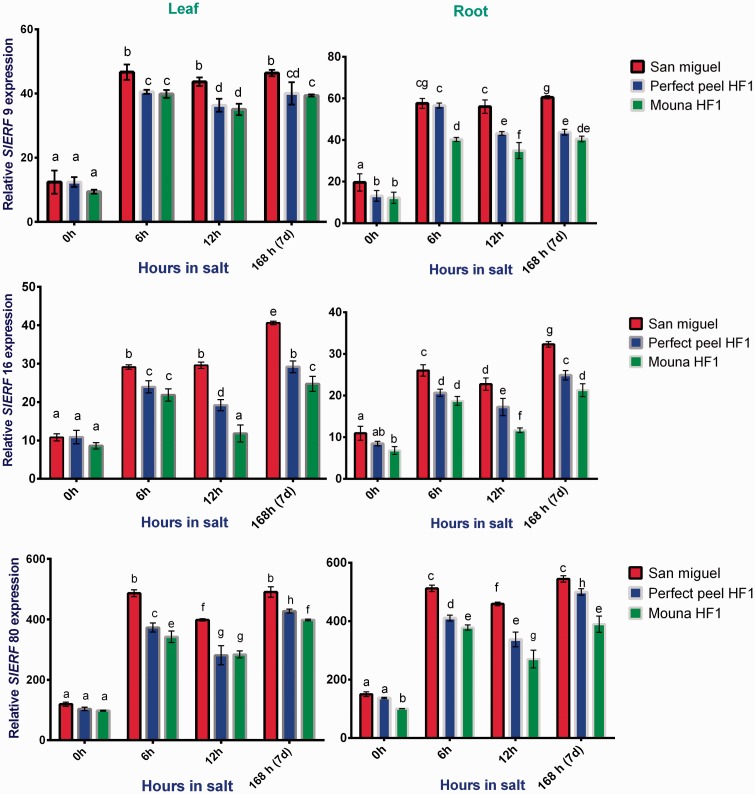
Relative gene expression of tomato *ERF9*, *ERF16* and *ERF80* in response to salt stress in leaves and roots San Miguel, Perfect peel HF1 and Mouna HF1genotypes. Total RNA was purified from tissues of tomato plants treated with 150 mM NaCl for 0 h, 6 h, 12 h and 7 days. Transcript level was analyzed by qRT-PCR using primers indicated in Table 1. Tomato *Actin* gene was used as reference gene. Error bars show the standard error between three replicates performed. Bars with different letters within each panel are significantly different at *P *> 0.05 according to Tukey's test.

Under salt stress, gene expression of *SIERF* family genes belonging to Group VI (*SIERF9* and *16*) exhibited the same pattern, increasing significantly in both leaves and roots and in all three genotypes. Two-way ANOVA followed by Tukey’s multiple comparisons test indicated that the relative *SIERF9*, 16 and *80* expression varied significantly among genotypes (leaves: *F*_2, 4 _=_ _17.93, *P* = 0.0101; roots: *F*_2, 4 _=_ _120.6, *P* = 0.0003); (leaves: *F*_2, 4 _=_ _310.6, *P* < 0.0001; roots: *F*_2, 4 _=_ _116.0, *P* = 0.0003) and (leaves: *F*_2, 4 _=_ _88.56, *P* = 0.0005; roots: *F*_2, 4 _=_ _241.3, *P* < 0.0001), respectively. Expression also changed significantly between stages of salt stress for *SIERF9*, *16* and *80* (leaves: *F*_3, 6 _=_ _744.2, *P* < 0.0001; roots: *F*_3, 6 _=_ _487.3, *P* < 0.0001), (leaves: *F*_3, 6 _=_ _300.8, *P* < 0.0001; roots: *F*_3, 6 _=_ _339.1, *P* < 0.0001) and (leaves: *F*_3, 6 _=_ _1335, *P* < 0.0001; roots: *F*_3, 6 _=_ _492.2, *P* < 0.0001), respectively. In addition, we observed significant genotypes × stages interaction terms (leaves: *F*_6, 12_= 3.388, *P* = 0.0342; roots: *F*_6, 12 _=_ _21.27, *P* < 0.0001), (leaves: *F*_6, 12 _=_ _23.22, *P* < 0.0001; roots: *F*_6, 12 _=_ _7.022, *P* = 0.0022) and (leaves: *F*_6, 12_= 19.26, *P* < 0.0001; roots: *F*_6, 12 _=_ _18.39, *P* < 0.0001) for *SIERF9*,*16* and *80*, respectively. Expression was high overall in the tolerant genotype compared with the other two genotypes. Transcripts of *SIERF80* accumulated in San Miguel at the first stage of treatment (6 h) to the same degree as in the latest stage (7 days) regardless of the tissue. These transcripts also increased significantly with Perfect Peel HF1 and Mouna HF1 genotypes (Fig. 8).

### *LeNHX* genes

**Figure 9.**
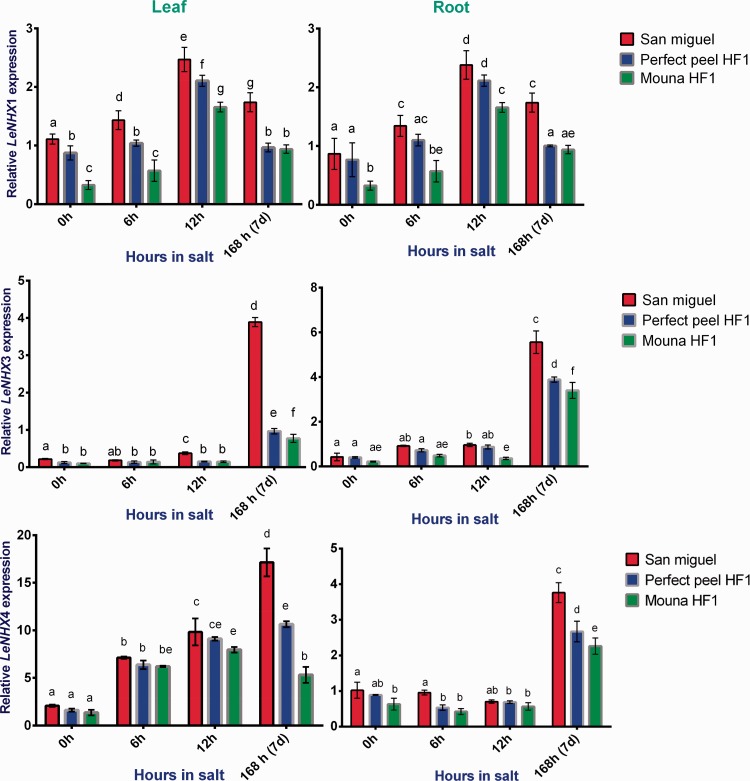
Relative gene expression of tomato *LeNHX1*, *LeNHX3* and *LeNHX4* in response to salt stress in in leaves and roots of San Miguel, Perfect peel HF1 and Mouna HF1 genotypes. Total RNA was purified from tissues of tomato plants treated with 150 mM NaCl for 0 h, 6 h, 12 h and 7 days. Transcript level was analyzed by qRT-PCR using primers indicated in Table 1. Tomato *Actin* gene was used as reference gene. Error bars show the standard error between three replicates performed. Bars with different letters within each panel are significantly different at *P* > 0.05 according to Tukey's test.

Results from two-way ANOVA indicated that stages of treatment had a significant overall effect on *LeNHX1*,*3* and *4* transcripts abundance (leaves: *F*_3, 6 _=_ _667.5, *P* < 0.0001; roots: *F*_3, 6 _=_ _106.8, *P* < 0.0001), (leaves: *F*_3, 6 _=_ _779.8, *P* < 0.0001; roots: *F*_3, 6 _=_ _1347, *P* < 0.0001) and (leaves: *F*_3, 6 _=_ _308.2, *P* < 0.0001; roots: *F*_3, 6 _=_ _613.6, *P* < 0.0001). Similarly results supported significant change in relative *LeNHX*1, 3 and 4 expression among genotypes (leaves: *F*_2, 4 _=_ _63.11, *P* = 0.0009; roots: *F*_2, 4 _=_ _236.3, *P* < 0.0001), (leaves: *F*_2, 4 _=_ _1542, *P* < 0.0001; *R*oots: *F*_2, 4 _=_ _74.41, *P* = 0.0007) and (leaves: *F*_2, 4 _=_ _232.3, *P* < 0.0001; roots: *F*_2, 4 _=_ _34.91, *P* = 0.0029), respectively. We detected significant genotypes × stages interaction terms (leaves: *F*_6,12 _=_ _3092, *P* < 0.0001; roots: *F*_6, 12 _=_ _15.02, *P* < 0.0001) and (leaves: *F*_6, 12 _=_ _38.01, *P* < 0.0001; roots: *F*_6, 12 _=_ _12.95, *P* = 0.0001) for *LeNHX3* and *4*, respectively, as well as for *LeNHX1* expression in leaves (*F*_6, 12 _=_ _5.219, *P* = 0.0074) but not in roots (*F*_6, 12 _=_ _2.090, *P* = 0.1306). In spite of a difference in the expression pattern of *LeNHX1*, *3* and *4*, the tolerant San Miguel genotype exhibited the highest expression of all isoforms at the end of the saline stress. *LeNHX1* showed an enhanced expression in both leaves and roots of stressed genotypes at 12 h post-salt treatment. *LeNHX3* transcripts were negligible during the first stage of salt treatment, whereas they increase significantly at the end. Salinity significantly enhanced *LeNHX4* expression in leaves and roots in all genotypes at 7 days of the stress imposition being highest in the tolerant genotype (Fig. 9).

### *HKT* Class I genes

**Figure 10.**
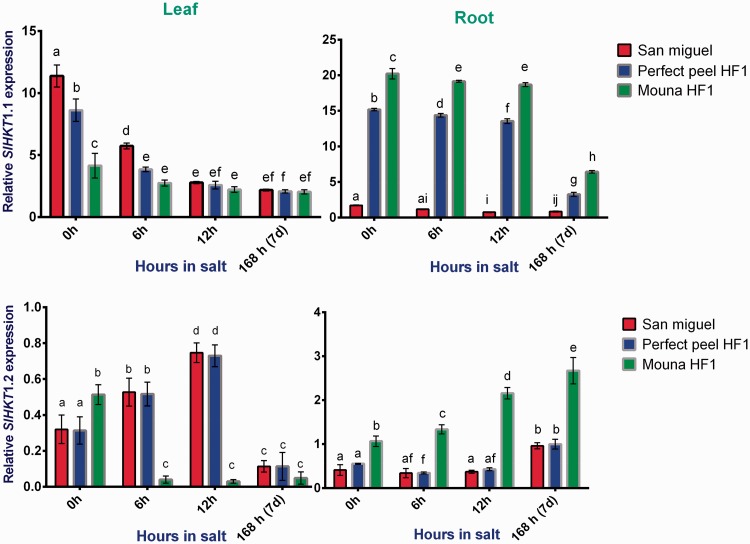
Relative gene expression of tomato *HKT1;1* and *HKT1;2* in response to salt stress in leaves and roots San Miguel, Perfect peel HF1 and Mouna HF1 genotypes. Total RNA was purified from tissues of tomato plants treated with 150 mM NaCl for 0 h, 6 h, 12 h and 7 days. Transcript level was analyzed by qRT-PCR using primers indicated in Table 1. Tomato *Actin* gene was used as reference gene. Error bars show the standard error between three replicates performed. Bars with different letters within each panel are significantly different at *P *> 0.05 according to Tukey's test.

Two way ANOVA followed by Tukey’s multiple comparisons test indicated that the relative *SIHKT* expression varied significantly between genotypes (leaves: *F*_2, 4 _=_ _124.1, *P* = 0.0003; roots: *F*_2, 4 _=_ _26 489, *P* < 0.0001) and (leaves: *F*_2, 4 _=_ _82.86, *P* = 0.0006; roots: *F*_2, 4 _=_ _221.4, *P* < 0.0001) and between stages of salt stress (leaves: *F*_3, 6 _=_ _343.7, *P* < 0.0001; roots: *F*_3, 6 _=_ _3520, *P* < 0.0001) and (leaves: *F*_3, 6 _=_ _55.95, *P* < 0.0001; roots: *F*_3, 6 _=_ _66.26, *P* < 0.0001) for *SIHKT1;1* and *1;2*, respectively. In addition, we observed significant interaction terms for these variables (leaves: *F*_6, 12 _=_ _29.57, *P* < 0.0001; roots: *F*_6, 12 _=_ _346.6, *P* < 0.0001) and (leaves: *F*_6, 12 _=_ _52.65, *P* < 0.0001; roots: *F*_6, 12 _=_ _60.86, *P* < 0.0001) for *SIHKT1;1* and *1;2*, respectively. *HKT1;1* was gradually down regulated in leaves during all stages of the salt stress treatment. Transcript level was negligible in roots of San Miguel whereas it seems significantly higher within Mouna HF1. Similarly, high salinity increased the level of *HKT1;2* transcripts in the roots of the sensitive genotype while remaining reduced during all the stress period within the tolerant and the mildly tolerant genotypes. In leaves, salinity clearly decreases *HKT1;2* expression at 7 days with all tested genotypes (Fig. 10).

## Discussion

Tomato plants, as sessile organisms, have evolved mechanisms that allow them to monitor their changing environment, as well as systems and strategies to react and adapt to these changes. Differences in sensitivity towards salt stress led to the classification of 15 local genotypes out of 20 as sensitive (Classes 3, 4 and 5). Therefore, salt stress seems to negatively affect tomato growth and would be expected to cause significant crop yield losses. The effects of such a stress may become obvious over weeks. In Tunisia, 25 % of the total of lands are salt-affected (FAO 2006). Tomato reaction to salt stress is carried through activating a stress response signal transduction network comprising physiological, biochemical, molecular and genetic changes.

### Na^+^, K^+^, Ca^2+ ^ and proline contents correlate with scale classes

Under salt stress, genotypes belonging to tolerant and mildly tolerant scale classes (1 and 3, respectively) displayed the lowest Na^+ ^ accumulation in leaves when compared to the sensitive class (5). San Miguel, the tolerant genotype, accumulated Na^+ ^ preferentially in roots while the sensitive Mouna HF1 accumulated more Na^+ ^ in leaves at the longest time post stress. This is likely due to the superior Na^+ ^ exclusion mechanism of genotypes clustered in either scale 1 or 3 classes (Dasgan *et al.* 2002). This finding is comparable with the way that potato cultivars respond to NaCl transport, since sensitive cultivars transport relatively more Na^+ ^ to leaves (Jaarsma *et al.* 2013). When exposed to saline conditions, plants show reduced uptake and lesser tissue retention of K^+ ^ (Munns *et al.* 2002; Chakraborty *et al.* 2012). Tolerant genotypes are able to maintain K^+ ^ homeostasis during all the salt stress stages (Shabala and Cuin 2008; Hauser and Horie 2010). Therefore, K^+ ^is considered as a key regulatory element in plant metabolic process by promoting Na^+ ^exclusion and osmotic adjustment (Chakraborty *et al.* 2016). In our experiments, leaves of tolerant and sensitive tomato genotypes behaved similarly, at the later stage of salt treatment, although the tolerant genotype accumulated significantly more K^+ ^ in its roots. Maintaining a low Na^+^/K^+ ^ ratio in tissues is critical for plant growth and metabolism under salty conditions (Wang *et al.* 2015). We found that leaf Na^+^/K^+ ^ ratios were correlated with salinity scale classes recorded during the first stage of salt stress. Indeed, tomato genotypes belonging to scale Classes 1 and 3 displayed higher Na^+^/K^+ ^ ratios than scale-Class 5 genotype at 12 h post-salt stress whereas this ratio did not fluctuate between genotypes either in leaves or in roots at the late stage of the stress. In roots, despite a peak recorded at 12 h post salt treatment for the mildly tolerant genotype, all genotypes showed slight variation for Na^+^/K^+ ^ ratios regardless of their scale class. Dasgan *et al.* (2002) reported that tomato genotypes with lower Na^+^/K^+ ^ ratios indicated lower scale classes with less salt damage. The long-term salt tolerance in tomato plants seems to be related to a lower leaf Na^+ ^ accumulation by reducing Na^+ ^ transport from root to shoot and a concomitant uniform cyotosolic K+  concentration maintaining thus leaf Na^+^/K^+ ^ homeostasis over time (García-Abellan *et al.* 2014; Wu *et al.* 2014). A comparison between wild type and mutant tomato plants showed the ability of the more tolerant salt plants to maintain their K^+ ^ content under moderate salt stress in roots. K^+ ^ level significantly declined leading to high Na^+^/K^+ ^ ratio in mutant tomato plants. Thus, the mutants were more sensitive to salt stress than the wild type. These changes can be attributed to a stronger ionic stress due to K^+ ^ loss from the root tissues (Poóra *et al.* 2015). Villalta *et al.* (2008) suggested that several genes located in chromosome 7 of tolerant tomato plants are responsible for governing the active mechanism of Na^+^/K ^+^  regulation.

When submitted to salt stress, another striking difference was observed between tomato genotypes. Tolerant scale Class 1 genotype exhibited an enrichment Ca^2+ ^ in leaf tissues. In addition to its effect on preventing Na^+ ^ entry into cells, Ca^2+ ^ is the most important universal signal carrier used by plants to convey information in many different cellular processes. Ca^2+ ^ seems to be necessary for maintenance of an appropriate K^+ ^ concentration in tissues (Subbarao *et al.* 1990). In addition, high Ca^2+ ^ has a beneficial effect by contributing to the maintenance of K^+ ^ uptake enhancing salt tolerant in tomato plants (Bacha *et al.* 2015).

The accumulation of ions requires the accumulation of solutes in the cytosol playing a role in both osmoprotection and osmotic adjustment under abiotic stress (Hasegawa *et al.* 2000; Flowers and Colmer 2008; Munns and Tester 2008). This accumulation of osmolytes, especially that of proline, is a common phenomenon in plants. Besides its role as an osmolyte, proline contributes to scavenging ROS, stabilizing subcellular structures, modulating cell redox homeostasis, supplying energy and functioning as a signal (Kavi-Kishor *et al.* 2005; Verbruggen and Hermans 2008; Szabados and Savouré 2010; Sharma *et al.* 2011). Although proline accumulation is a common response to salt stress in tomato, the extent of its accumulation varies between tolerant and sensitive genotypes. Indeed, our findings revealed that proline accumulation increases greatly within the tolerant genotype, mainly in leaves and when compared to the most sensitive genotype. Proline is accumulated preferentially in leaves in order to maintain chlorophyll level and cell turgor to protect photosynthetic activity under salt stress (Silva-Ortega *et al.* 2008). Proline has also a potential role in scavenging ROS products (Soshinkova *et al.* 2013).The accumulation of proline in plants under stress is caused either by the induction of expression of proline biosynthesis genes (*P5CS, P5CR*) or by the repression of the genes of its degradation pathway (PDH silencing) (Marco *et al.* 2015).

### Salt stress-induced up-regulation of antioxidant enzymes

The increases in CAT, APX and GPOX activities are an adaptive trait to overcome salt damage by reducing toxic levels of H_2_O_2_ and provide protection against oxidative stress (Sudhakar *et al.* 2001; Bor *et al.* 2003; Mittova *et al.* 2003; Chawla *et al.* 2013). In our study, salt stress modulates the responses of antioxidative enzymes in both leaves and roots according to the tested genotype and the period of stress imposition. Oxidative stress defence occurs in the tolerant San Miguel genotype through an increase in APX and GPOX activities especially in roots and leaves. CAT, APX and GPOX have been reported as antioxidant enzymes in different plant tissues (Chawla *et al.* 2013). CAT is often related to an enhanced tolerance to salt stress (Mittova *et al.* 2004; Gao *et al.* 2008). Similarly, APX activity under salinity stress increases (Gossett *et al.* 1994; Hernàndez *et al.* 2000; Lee *et al.* 2001; Mittova *et al.* 2004). Within root organelles of salt-tolerant genotypes of tomato, the increase in APX activity was higher than that of SOD under salt stress. This finding indicates that under salinity, the rate of H_2_O_2_ detoxification is higher than that of its production leading to alleviation of oxidative stress. Similarly, decreased H_2_O_2_ and lipid peroxidation levels were found in peroxisomes of salt-treated tolerant plants. These responses to salinity were the result of differentially increased activities of APX and CAT over that of SOD (Mittova *et al.* 2004). An improved stress tolerance has been observed in several transgenic plants over-expressing antioxidant enzymes such as GPOX and APX (John *et al.* 2010). Kim *et al.* (2014) reported a positive response of GPOX to environmental stimuli such as salt stress enhancing tolerance of *Panax ginseng* plants against abiotic stresses. APX activity was clearly enhanced in the salt-tolerant *L. pennellii* (Mittova *et al.* 2015). Overall, our findings demonstrate that inherent activities of the isozymes are present in tomato leaf and root but are expressed differentially between genotypes. These variations reflect differences in both tissues- and species-dependent expression of these isozymes (Mittova *et al.* 2015).

### Expression analysis of transporters *(HKT* Class 1 and *LeNHX)*

Many plants have developed an efficient method to keep Na^+ ^ concentration in the cytoplasm at a low level (Gupta and Huang 2014). The data in this work showed that leaf expression of *HKT1.2* was high and similar between San Miguel and Perfect peel HF1 during the first stage of salt treatment before reducing drastically and similarly in both genotypes at the later stage. At the same time, leaf expression of *HKT1;1* decreased gradually in leaves regardless of the tested genotype. Overall, root expression of *HKT1;1* and *HKT1;2* was significantly reduced in the tolerant genotype compared with the most sensitive one. The differences observed in the expression levels of *HKT1* genes in tomato genotypes are probably linked to the contribution of each allele to Na^+ ^ movement and tissue content. These findings are in accord with those published by Almeida *et al.* (2013) since they found a positive relation between *HKT1;1* and *HKT1;2* expression and Na^+ ^ content in leaves but not in roots. Interestingly, in *Arabidopsis*, the salt tolerance of ecotypes adapted to coastal and saline soils is associated with high leaf Na^+ ^ concentration due to a weak expression of the *AtHKT1;1* allele in roots (Rus *et al.* 2006; Baxter *et al.* 2010).

In addition to HKT, LeNHX cation/H^+ ^ antiporters contribute to the sequestration of Na^+ ^ in vacuoles, the regulation of the homeostasis of K^+ ^ and endosomal pH regulation under normal and saline conditions (Barragán *et al.* 2012; Bassil *et al.* 2012; Leidi *et al.* 2010). We involved in our study three different isoforms corresponding to *LeNHX1*, *LeNHX3* and *LeNHX4*. LeNHX1 is a tonoplast localized protein mediating K^+ ^ uptake at the tonoplast, for turgor regulation and stomatal function (Barragán *et al.* 2012). LeNHX3 and LeNHX4 are involved in Na^+^, K^+ ^ and H^+ ^ homeostasis (Gàlvez *et al.* 2012). *LeNHX3* seems also to be linked to a QTL for Na^+ ^ leaf concentration (Villalta *et al.* 2007; 2008). In our study, these three isoforms were differentially expressed allowing the discrimination between tolerant and sensitive genotypes. *LeNHX1* showed a low expression in normal conditions and rapidly increases during the early stage of stress imposition, then it decreased at the latest stage (at 7 days). Expression of *LeNHX1* seems to be correlated with low accumulation of Na+  in leaves and high accumulation in roots of the tolerant genotype. *LeNHX3* and *LeNHX4* were highly expressed within San Miguel, especially during the latest period of the treatment. This pattern is associated with a reduced Na^+ ^ content in leaf tissue and a high accumulation in roots in line with previous studies (Venema *et al.* 2003; Almeida *et al.* 2014). Either in the absence of stress or during the early stage of salt stress, *LeNHX3* expression remained drastically reduced in both leaf and root tissues. This expression increased later, especially in the tolerant genotype. This may be due to enhanced cellular Na^+ ^ concentration as described by Gàlvez *et al.* (2012). Apart from *LeNHX3*, *LeNHX4* showed also basal expression level in leaves in the absence of salt stress that increased 7 days post-treatment particularly in the tolerant genotype. This agreed with other-published data showing that *LeNHX4* and closely related isoforms *in Arabidopsis* are rapidly induced by salt stress in roots and especially in leaves (Gàlvez *et al.* 2012; Pardo *et al.* 2006).

### Expression analysis of *WRKY* and *ERF* genes

In response to salinity stress, a large number of salt-responsive transcription factors and genes, being either upregulated or downregulated, have been identified and characterized using transcriptomic and genomic approaches (Bakshi and Oelmüller 2014). In this present work, transcription of *SIWRKY8, SIWRKY31* and *SIWRKY*39 was induced with a similar a pattern of expression between leaf and root tissues. *SIWRKY8 and SIWRKY31* showed abundant transcripts accumulation particularly within the tolerant genotype at all times of the treatment and even in the absence of the salt stress. In contrast, *SIWRKY*39 was highly and similarly expressed within sensitive and tolerant genotypes at the beginning of the stress (12 h) then showing a significant increase in the tolerant genotype (7 days). These transcription factors were reported to be up-regulated by salt stress (Huang *et al.* 2012). Thus, 81 *WRKY* genes were reported to display constitutive or induced expression patterns which are tomato tissue-specific. The majority of the *WRKY* gene family as well as their orthologs in *Arabidopsis* showed up-regulation under stress (Jiang and Deyholos 2009; Liu *et al.* 2011).

Besides members of *WRKY* gene family, *ERF* transcription factors are the most important regulators modulating gene expressions (Sharma *et al.* 2010). Our study indicated that rapid and high expression of *SIERF9*, *16* and *80* is closely connected to salt tolerance during all the stages of the salt stress treatment. ln line with such findings, Sharma *et al.* (2010) showed that *SIERF80* was 400-foldes up-regulated during salt stress. They also provided evidence that over expressing of *SlERF5* in transgenic tomato plants leads to an increased resistance to salt and drought stress.

## Conclusion

Salinity is one of the major abiotic stresses world-wide, particularly in Tunisia due to the soil salinization and the poor quality of water irrigation. Salinity severely limits yields, threatening land productivity in arid and semi-arid areas leading to food imbalance of these regions. The ability to face abiotic challenges involves a complex of responses at the whole plant level. Responses are themselves part of effective ways to improve and protect tomato crops from the adverse effects of soil salinization. This is the first study investigating phenotypical, physiological and molecular responses of Tunisian tomato genotypes to salt stress. Associations were pointed out between exhibited phenotypes, ion and proline accumulation, APX, CAT and GPX activities and gene expression. Salt tolerance seems to be related to a lower leaf accumulation in the long term by reducing Na^+ ^ transport from root to leaves. Besides, accumulation of proline was found to be linked to tolerance being much higher within tolerant genotype. As production of ROS generated by salt stress is always enhanced, APX, Cat and GPOX activities were stimulated mainly in the tolerant genotype at the later stage of treatment. The described expression pattern of allelic genes belonging to *WRKY* (8, 31 and 39), *ERF* (9, 16 and 80), *LeNHX* (1, 3 and 4) and *HKT* (Class 1) families support the view that they are involved in mechanisms associated with a response to salt stress and can be considered as markers to be used in discriminating tomato genotypes. Data generated from this study will be helpful in selecting candidate genotypes to be used by growers in coastal areas or as progenitors in breeding programmes.

## Sources of Funding

This work was partially supported by the Ministry of Higher Education and Scientific Research of Tunisia and carried out within the USAID-MERC Project TA-MOU-08-M28-048.

## Contributions by the Authors

C.G., H.F. and F.G. conceived and designed the experiments. C.G. performed the experiments. C.G., H.F. and F.G. analysed the data. F.G. wrote the paper with assistance from C.G and D.G.

## Conflict of Interest Statement

None declared.

## Supplementary Material

Supplementary Data

## Supporting Information

The following additional information is available in the online version of this article —

**Figure S1.** Seedlings grown in Hoagland solution to half-strength in jars with continuous aeration. Plants with four fully developed true leaves were transferred into plastic pots containing a mixture of peat and sand, then irrigated with one-half Hoagland solution added with 150 mM NaCl (15 dS/m, pH 7.5).

**Figure S2**. The salinity scale classes used in the experiment according to Dasgan *et al.* (2002). 1/normal green plants with or without slight inward curly leaves; 2/green plants with complete inward curly leaves; 3/addition to complete curly leaves, dry leaves from moderate to severe damages; 4/most leaves with drying damages; 5/all leaves of the plant with drying damages.

**Figure S3.** Data relative to dry weight of leaves and roots. Data, expressed as g DW per plant, are means SD of three independent experiments, using 10 plants per genotype and per treatment. Bars with different letters within each panel are significantly different at P > 0.05 according to Tukey's test.

**Figure S4.** Stability of tomato *Actin* gene expression (expressed as cycle threshold- (Cts-)) in response to salt stress treatment in San Miguel, Perfect peel HF1 and Mouna HF1 genotypes. Total RNA was purified from leaf and root of tomato plants treated with 150 mM NaCl for 0 h, 6 h, 12 h and 7 days. Transcript level was analyzed by qRT-PCR using primers indicated in Table 1. The data reported are the mean ± SE of three values.

**Figure S5.** Expression profiles of candidate genes. The results of the relative expression levels (Δct) of candidate genes under salt stress treatments (6 h, 12 h and 7 days) compared to the controls were used for hierarchical cluster analysis with Data Assist v3.01. The colour scale represents relative expression levels, with red as increased transcript abundance and green as decreased transcript abundance.
