# Supplementary material for: Effect of salt stress on ion concentration, proline content, antioxidant enzyme activities and gene expression in tomato cultivars
Source: AoB Plants. 2016 Oct 26;8:plw055. doi: 10.1093/aobpla/plw055 (PMC5091694; doi:10.1093/aobpla/plw055)
Supplement: Supplementary Data [file supp_8_plw055_index.html]

Effect of salt stress on ion concentration, proline content, antioxidant enzyme activities and gene expression in tomato cultivars — Supplementary Data 

# Effect of salt stress on ion concentration, proline content, antioxidant enzyme activities and gene expression in tomato cultivars

## Supplementary Data

files

- Supplementary Data - docx file
