# Supplementary material for: Effect of salt stress on ion concentration, proline content, antioxidant enzyme activities and gene expression in tomato cultivars
Source: AoB Plants. 2016 Oct 26;8:plw055. doi: 10.1093/aobpla/plw055 (PMC5091694; doi:10.1093/aobpla/plw055)
Supplement: Supplementary Data [file supp_plw055_aobplants-16046-s01.docx]

**Figure S1.** Seedlings grown in Hoagland solution to half-strength in jars with continuous aeration. Plants with four fully developed true leaves were transferred into plastic pots containing a mixture of peat and sand, then irrigated with one half Hoagland solution added with 150 mMNaCl (15 dS/m, pH 7.5).

**
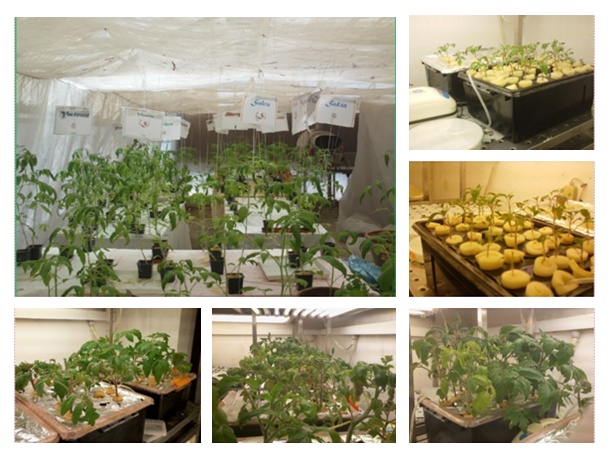
**

**Figure S2.** The salinity scale classes used in the experiment according to Dasgan et al., (2002). 1/ normal green plants with or without slight inward curly leaves; 2/ green plants with complete inward curly leaves; 3/ addition to complete curly leaves, dry leaves from moderate to severe damages; 4/ most leaves with drying damages; 5/ all leaves of the plant with drying damages.

**
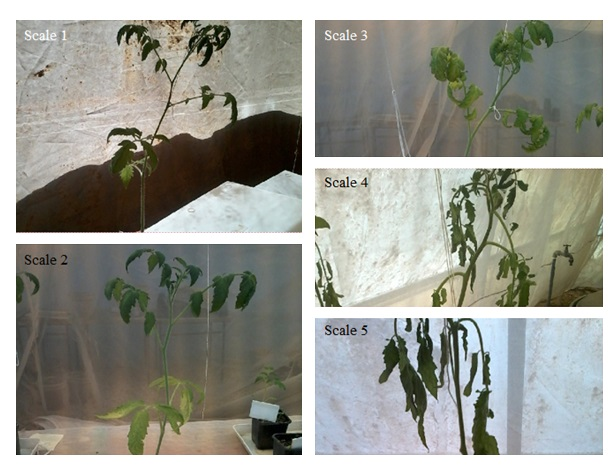
**

**Figure S3.** Data relative to dry weight of leaves and roots. Data, expressed as g DW per plant, are means SD of three independent experiments, using 10 plants per genotype and per treatment. Bars with different letters within each panel are significantly different at P>0.05 according to Tukey's test.

**
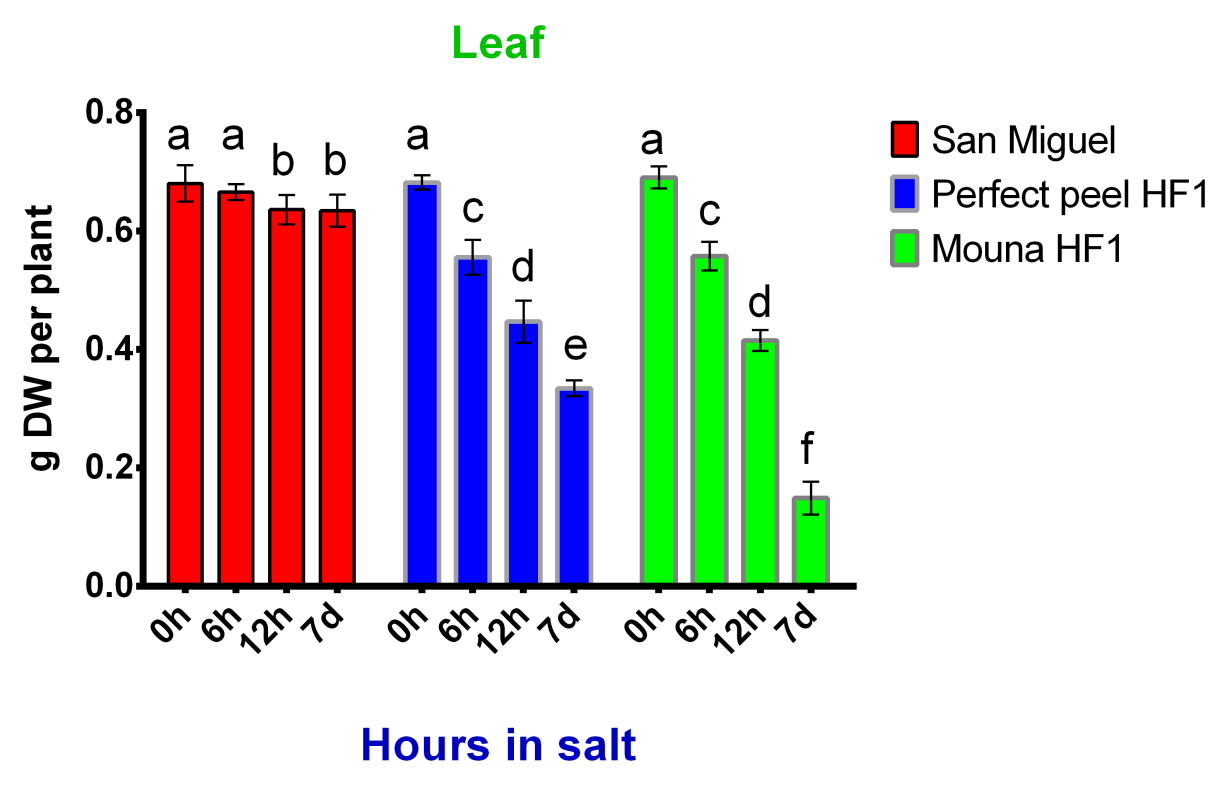
**

**
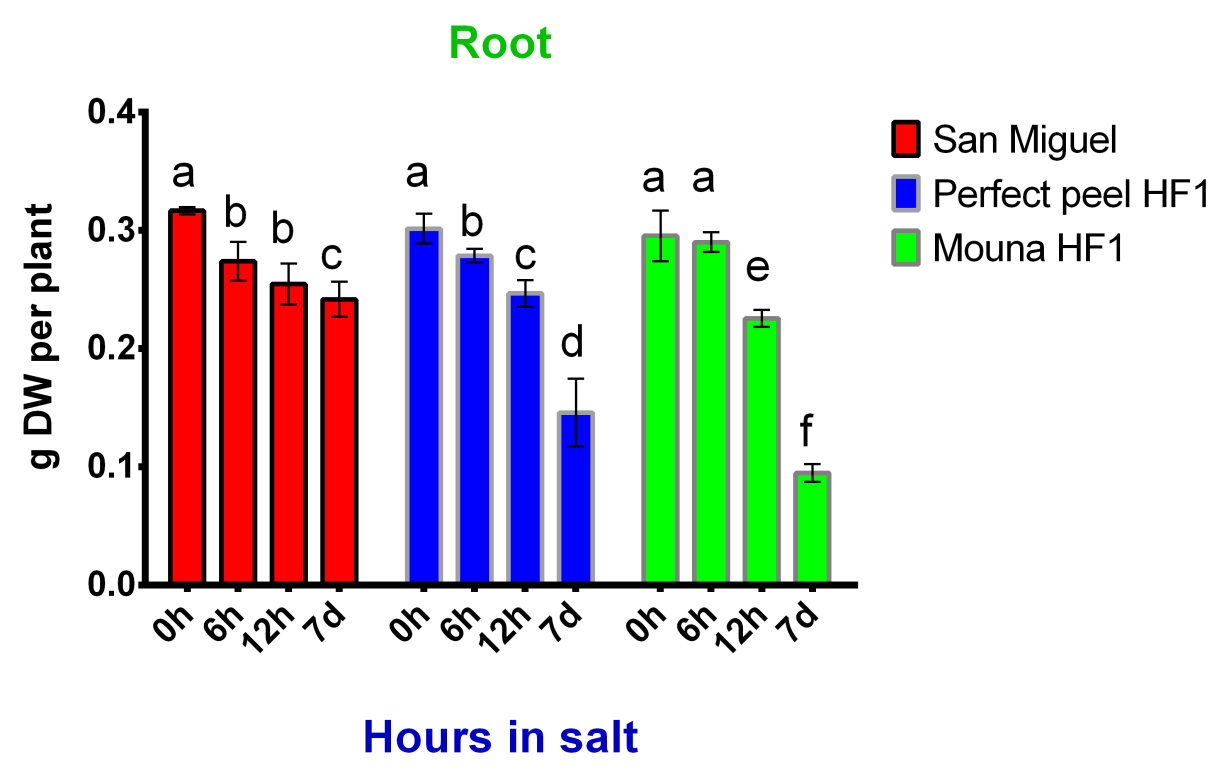
**

**Figure S4.** Stability of tomato *Actin* gene expression (expressed as cycle threshold- Cts-) in response to salt stress treatment in San Miguel F1, Perfect peel HF1 and Mouna HF1 genotypes. Total RNA was purified from leaf and root of tomato plants treated with 100 mM NaCl for 0h, 6h, 12h and 7 days. Transcript level was analyzed by qRT-PCR using primers indicated in Table 1. The data reported are the mean ± SE of 3 values.**

**

**Figure S5.** Expression profiles of candidate genes. The results of the relative expression levels (delta ct) of candidate genes under salt stress treatments (6h, 12h and 7days) compared to the controls were used for hierarchical cluster analysis with Data Assist v3.01. The color scale represents relative expression levels, with red as increased transcript abundance and green as decreased transcript abundance.


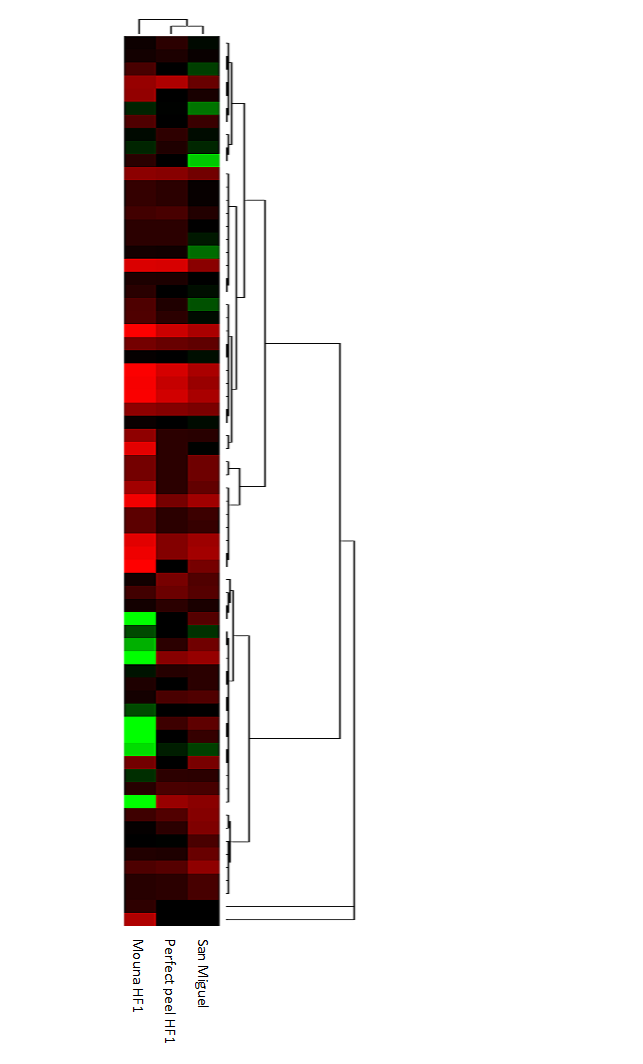


Mouna HF1

Perfect peel HF1

San Miguel
